# Efficacy of Plant‐Derived Therapies for Primary Dysmenorrhea: A Systematic Review and Meta‐Analysis of Randomized Controlled Trials

**DOI:** 10.1002/ptr.70324

**Published:** 2026-04-15

**Authors:** Lifei Wu, Inmaculada Xu Lou, Zhenting Hu, Gan Wang, Sammit Vishram Deshpande, Rocío Cáceres‐Matos

**Affiliations:** ^1^ Zhejiang Chinese Medical University Hangzhou China; ^2^ Department of Nursing, Faculty of Nursing, Physiotherapy and Podiatry University of Seville Seville Spain; ^3^ Research Group CTS‐1050: Complex Care, Chronicity and Outcomes in Health Seville Spain

**Keywords:** herbal medicine, menstrual pain, plant‐based, primary dysmenorrhea, traditional medicine

## Abstract

Primary dysmenorrhea is defined as recurrent, cramp‐like lower abdominal pain occurring during menstruation in the absence of underlying pelvic pathology. A significant proportion of the population opts for natural plant‐based remedies due to their perceived lower incidence of adverse effects. This study aims to evaluate the efficacy of plant‐based interventions in the management of primary dysmenorrhea. Systematic review and meta‐analysis. A comprehensive search was conducted across five databases (PubMed, Web of Science, Medline, Embase, and Scopus) covering the period from 1990 to 2025. Randomized controlled trials (RCTs) investigating the efficacy of plant‐based products or herbal medicine for the treatment of primary dysmenorrhea were included. Searches, data extraction, and risk of bias assessment were performed independently by two reviewers. Discrepancies were resolved through consultation with a third reviewer expert in the topic. A total of 47 studies were included in the systematic review, of which 22 were incorporated into the meta‐analysis. The findings indicate that herbal medicine can be as effective as conventional pharmacological treatments (*n* = 1133, MD: 0.12, 95% CI: −0.29 to 0.53, *p* = 0.58, *I* ^2^ = 95%), such as mefenamic acid or ibuprofen, in managing primary dysmenorrhea. Additionally, a statistically significant difference was observed when compared to placebo (*n* = 941, MD: −1.83, 95% CI: −2.32 to −1.34, *p* < 0.00001, *I* ^2^ = 92%). Adverse effects were infrequent, mild in severity, and resolved upon discontinuation of the intervention. Overall, the certainty of evidence is low to very low due to high heterogeneity and moderate risk of bias. Plant‐based treatments may reduce pain associated with primary dysmenorrhea and are probably comparable to conventional drug therapies. However, further studies employing more rigorous methodologies are required to establish stronger evidence regarding the efficacy and safety of herbal medicine in addressing this health condition.

## 1 Introduction

Primary dysmenorrhea, characterized by recurrent pelvic cramping pain during menstruation in the absence of underlying pathology, is one of the most prevalent gynecological disorders worldwide (Del Prado et al. 2025). Its prevalence varies across different populations, but it is estimated to affect between 45% and 90% of women of reproductive age (Wang and Zhu 2025).

This condition primarily affects women of reproductive age, particularly younger individuals, with a higher prevalence in those under 25, especially during the early years following menarche (Del Prado et al. 2025). Generally, primary dysmenorrhea tends to decrease with age and after childbirth, although some women continue to experience symptoms throughout their reproductive years (Wang and Zhu 2025).

The primary symptoms associated with primary dysmenorrhea include lower abdominal pain, lower back pain, nausea, fatigue, headache, diarrhea or constipation, and mood changes. These symptoms typically begin 6 to 12 h before menstruation and may persist for 48–72 h (Liu et al. 2025). The condition significantly impacts quality of life, contributing to absenteeism, reduced productivity, and psychological distress (Del Prado et al. 2025). Conventional management primarily involves NSAIDs (nonsteroidal anti‐inflammatory drugs) and hormonal contraceptives, which, while effective for many, are associated with various adverse effects (Taniguchi et al. 2020; Leem et al. 2019).

Herbal remedies have long been integral to traditional medical systems, such as Ayurveda and Traditional Chinese Medicine, and are frequently used to alleviate menstrual pain and regulate menstrual cycles (Chen et al. 2014; Flower et al. 2011). Recent studies suggest that phytochemicals in herbs such as ginger, cinnamon, turmeric, ashwagandha, and chamomile may modulate inflammatory pathways, prostaglandin synthesis, and uterine hypercontractility, all of which are involved in the development of menstrual pain (Afiat et al. 2024; Xu et al. 2020). Due to the high rates of women that suffer from primary dysmenorrhea and the disturbances generated by it, more research regarding it is necessary. Therefore, this systematic review and meta‐analysis aims to evaluate the efficacy of plant‐based interventions in the management of primary dysmenorrhea.

## 2 Materials and Methods

This systematic review and meta‐analysis on the efficacy of plant‐based products or traditional medicine for primary dysmenorrhea was conducted following the PRISMA (Preferred Reporting Items for Systematic Reviews and Meta‐Analyses) guidelines (Page et al. 2021) and was registered in PROSPERO (International Prospective Register of Systematic Reviews) (registration number: CRD420251000080).

### 2.1 Search Strategy and Data Sources

A systematic search was conducted in five international databases: PubMed, Web of Science (WOS), Medline, Embase, and Scopus. Additionally, ClinicalTrials.gov and Google Scholar were consulted to mitigate potential publication bias. The following search terms were used: (“Dysmenorrhea” OR “Dysmenorrheas” OR “Menstruation, Painful” OR “Menstruations, Painful” OR “Painful Menstruation” OR “Painful Menstruations” OR “Pain, Menstrual” OR “Menstrual Pain” OR “Menstrual Pains” OR “Pains, Menstrual”). No plant‐related terms were included to avoid omitting relevant information. Searches were conducted during the months of January and February 2025.

### 2.2 Study Selection Criteria

#### 2.2.1 Types of Studies

Only randomized controlled trials (RCTs) published in English and/or Chinese between January 1990 and February 2025 were included.

#### 2.2.2 Types of Participants

Studies involving women of reproductive age, from menarche to menopause, diagnosed with primary dysmenorrhea were included, with no restrictions on ethnicity or geographical origin. Studies on secondary dysmenorrhea were excluded unless it was analyzed separately from primary dysmenorrhea. Studies that did not specify the type of dysmenorrhea were also excluded to mitigate bias.

#### 2.2.3 Types of Interventions

Studies were included if the intervention involved the exclusive use of plant‐based products, herbal extracts, or plant‐based traditional medicine for the management of primary dysmenorrhea, including formulations such as capsules, oils, or pills. External‐use products were excluded. No restrictions were placed on the dosage or duration of the intervention. Other traditional medicine methods such as exercise, moxibustion, acupuncture, cupping, or aromatherapy were also excluded. Studies that did not specify the dosage or composition of the intervention were excluded, as well as those using nutritional supplements without indicating their plant‐based origin.

#### 2.2.4 Types of Comparisons

Studies in which the control group received a placebo or conventional pharmacological treatment were included.

#### 2.2.5 Outcome Measures

The primary outcome of this study is the reduction in the intensity and/or duration of menstrual pain. Secondary outcomes include the reduction of associated symptoms such as nausea, vomiting, headache, and fatigue. Additionally, the study aims to evaluate the improvement in dysmenorrhea‐related quality of life and to assess any reported adverse effects. The measurement tools to assess pain included the VAS (Visual Analog Scale), McGill Pain Scale (McGill Pain Questionnaire), NRS (Numeric Rating Scale), TOTPAR (Total Pain Relief), PDAF (Pain Disability Assessment Form), and VMSS (Verbal Multidimensional Scoring System).

### 2.3 Data Extraction

Two independent reviewers examined the selected articles and extracted data according to predefined criteria, collecting information on participants, interventions, outcome measures, and study findings. Discrepancies were resolved through discussion with a third author acting as an impartial evaluator.

The data extraction table includes the following variables: (1) author's name; (2) year of publication; (3) country of origin; (4) study design; (5) sample size; (6) severity of dysmenorrhea; (7) study duration; (8) participant age; (9) type of intervention; (10) type of control (placebo or conventional drug); (11) measurement tools; (12) primary outcomes (pain intensity and pain duration); (13) secondary outcomes; and (14) adverse effects.

### 2.4 Methodological Quality and Risk‐of‐Bias (RoB) Assessment

RoB was assessed using the Cochrane RoB 2.0 tool for randomized trials (Higgins et al. 2011). This tool evaluates five domains: (1) randomization process, (2) deviations from intended interventions, (3) missing outcome data, (4) measurement of the outcome, and (5) selection of the reported result. Each domain was judged as “Low risk,” “Some concerns,” or “High risk” of bias. Discrepancies between reviewers were resolved through discussion.

To assess the quality of the evidence and the strength of the recommendation, the GRADE system (Grading of Recommendations, Assessment, Development, and Evaluation) was applied using the GRADEpro GDT software, evaluating only the studies included in the meta‐analysis.

### 2.5 Data Synthesis

In the meta‐analysis, only studies with no high RoB in any of the criteria assessed using the RoB 2.0 tool, and pain outcomes measured using the VAS and expressed as mean ± standard deviation (mean ± SD) were included. We used the Cochrane Collaboration's software program, Review Manager (RevMan), version 5.3 for Windows (Copenhagen, The Nordic Cochrane Center, Copenhagen, Denmark) to conduct all statistical analyses. Differences between the intervention and control groups were calculated. To analyze the mean difference (MD) with 95% confidence intervals (CIs) between both groups, the generic inverse variance method in RevMan was applied. MD was used because all the included studies measured the same outcome using the same measurement tool. The data were then pooled across the studies using random‐effects models when appropriate. The *I* ^2^ test was used to assess the heterogeneity of the included studies. We also intended to conduct subgroup analyses according to the different types of interventions or controls. To assess publication bias, a funnel plot was generated, and a leave‐one‐out sensitivity analysis was performed to evaluate the robustness of the results.

## 3 Results

### 3.1 Description of the Included Studies

**FIGURE 1:**
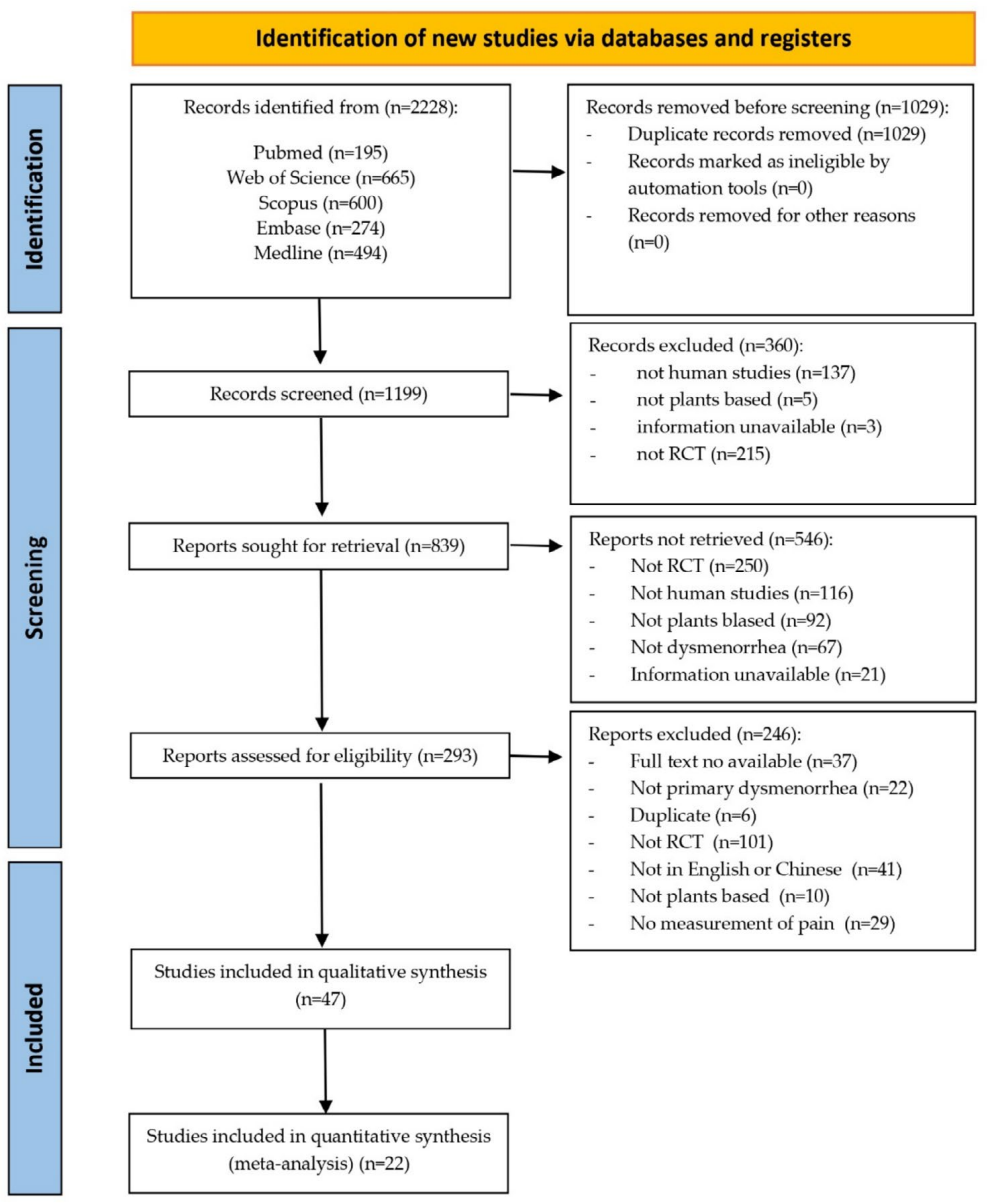
Flow diagram of the selection process.

A total of 2228 potential articles were identified, of which only 47 met the inclusion criteria (Figure 1). The characteristics of the included studies are summarized in Table 1 (Shirvani et al. 2015; Sardashti et al. 2020; Rahnama et al. 2012; Jahangirifar et al. 2018; Abadian et al. 2016; Dehkordi et al. 2019; Rezaeyan et al. 2015; Modaress Nejad and Asadipour 2006; Vannabhum et al. 2016; Nuha et al. 2023; Mirabi et al. 2011; Safdari‐Dehcheshmehi and Parvin 2016; Bokaie et al. 2013; Masoumi et al. 2016; Zeraati et al. 2014). Among these, one study was conducted in China (Chai et al. 2020), one in Egypt (Abd‐El‐Maeboud et al. 2014), four in India (Agarwal and Chaudhary 2023; Anjum and Sultana 2019; Banu et al. 2025; Rehman et al. 2015), one in Indonesia (Nuha et al. 2023), 34 in Iran (Shirvani et al. 2015; Sardashti et al. 2020; Rahnama et al. 2012; Jahangirifar et al. 2018; Abadian et al. 2016; Dehkordi et al. 2019; Rezaeyan et al. 2015; Modaress Nejad and Asadipour 2006; Mirabi et al. 2011, 2017; Bokaie et al. 2013; Masoumi et al. 2016; Aboualsoltani et al. 2024; Atallahi et al. 2014; Bani et al. 2014; Behmanesh et al. 2019; Direkvand‐Moghadam and Khosravi 2012; Falahieh et al. 2019; Golkhatmy et al. 2024; Heidarifar et al. 2014; Hesami et al. 2021; Jaafarpour et al. 2015; Jafari et al. 2019; Jenabi 2013; Jenabi and Fereidoony 2015; Kashefi et al. 2014; Nahid et al. 2009; Rad et al. 2018; Shabani et al. 2022; Tabari et al. 2020), one in Jamaica (Fletcher et al. 2013), one in Mexico (Doubova et al. 2007), three in Thailand (Vannabhum et al. 2016; Leemud et al. 2024; Sriyakul et al. 2012), and one in Turkey (Amanak 2020). Five studies employed a crossover design (Dehkordi et al. 2019; Rezaeyan et al. 2015; Masoumi et al. 2016; Abd‐El‐Maeboud et al. 2014; Rad et al. 2018), while 28 were double‐blind trials (Rahnama et al. 2012; Jahangirifar et al. 2018; Dehkordi et al. 2019; Modaress Nejad and Asadipour 2006; Vannabhum et al. 2016; Mirabi et al. 2011, 2017; Masoumi et al. 2016; Zeraati et al. 2014; Chai et al. 2020; Agarwal and Chaudhary 2023; Banu et al. 2025; Aboualsoltani et al. 2024; Bani et al. 2014; Behmanesh et al. 2019; Falahieh et al. 2019; Golkhatmy et al. 2024; Heidarifar et al. 2014; Hesami et al. 2021; Jaafarpour et al. 2015; Jenabi and Fereidoony 2015; Nahid et al. 2009; Shabani et al. 2022; Tabari et al. 2020; Fletcher et al. 2013; Doubova et al. 2007; Sriyakul et al. 2012; Younesy et al. 2014). The most commonly used intervention duration was two menstrual cycles, reported in 29 studies (Jahangirifar et al. 2018; Abadian et al. 2016; Bokaie et al. 2013; Anjum and Sultana 2019; Banu et al. 2025; Aboualsoltani et al. 2024; Atallahi et al. 2014; Bani et al. 2014; Behmanesh et al. 2019; Direkvand‐Moghadam and Khosravi 2012; Falahieh et al. 2019; Golkhatmy et al. 2024; Heidarifar et al. 2014; Hesami et al. 2021; Jafari et al. 2019). The plant‐based interventions were categorized into three groups: (i) Herbal medicine: traditional medicine formulas (Vannabhum et al. 2016; Chai et al. 2020; Nahid et al. 2009; Leemud et al. 2024; Sriyakul et al. 2012); (ii) Standardized phytomedicines: *Achillea millefolium* (Jenabi and Fereidoony 2015), *Trachyspermum ammi L*. (Zali et al. 2023), *Aloe vera* (Sardashti et al. 2020), *Anethum graveolens* (Heidarifar et al. 2014), *Cassia fistula Linn* and *Myristica fragrans* Houtt (Anjum and Sultana 2019), *Chamomilla recutita* (Shabani et al. 2022), *Cinnamomum verum* (Jahangirifar et al. 2018; Jaafarpour et al. 2015), *Citrus aurantium* L. (Aboualsoltani et al. 2024), *Eryngium caucasicum* Trautv. (Behmanesh et al. 2019), *Foeniculum vulgare* (Modaress Nejad and Asadipour 2006; Bokaie et al. 2013; Zeraati et al. 2014), *Trigonella foenum‐graecum* (Younesy et al. 2014), *Zingiber officinale* (Shirvani et al. 2015; Rahnama et al. 2012; Jenabi 2013; Kashefi et al. 2014; Rad et al. 2018), *Glycyrrhiza glabra* L. (Jafari et al. 2019), *Juniperus communis* L. (Banu et al. 2025), *Melissa officinalis* (Safdari‐Dehcheshmehi and Parvin 2016; Mirabi et al. 2017), *Morinda citrifolia* (Fletcher et al. 2013), *Nigella sativa* (Falahieh et al. 2019), *Mentha piperita* (Masoumi et al. 2016), *Psidium guajava* folium (Doubova et al. 2007), *Rheum rhabarbarum* (Rehman et al. 2015), *Rosa damascena* (Bani et al. 2014), *Rosmarinus officinalis* (Golkhatmy et al. 2024), *Salix* spp. (Dehkordi et al. 2019), *Thymus vulgaris Shirazi* (Direkvand‐Moghadam and Khosravi 2012), *Teucrium polium* (Abadian et al. 2016), *Curcuma longa* (Agarwal and Chaudhary 2023; Hesami et al. 2021; Tabari et al. 2020), *Xysmalobium undulatum* (Abd‐El‐Maeboud et al. 2014), and *Valeriana officinalis* (Mirabi et al. 2011); and (iii) Food/nutrition‐based interventions: cacao derivatives such as dark chocolate (Nuha et al. 2023), *Ficus carica* (Amanak 2020), olive oil (Rezaeyan et al. 2015), and wheat germ (Atallahi et al. 2014). A total of 33 studies compared the intervention with a pharmaceutical drug (Shirvani et al. 2015; Sardashti et al. 2020; Abadian et al. 2016; Dehkordi et al. 2019; Rezaeyan et al. 2015; Modaress Nejad and Asadipour 2006; Nuha et al. 2023; Safdari‐Dehcheshmehi and Parvin 2016; Bokaie et al. 2013; Masoumi et al. 2016; Zeraati et al. 2014; Abd‐El‐Maeboud et al. 2014; Anjum and Sultana 2019; Banu et al. 2025; Rehman et al. 2015; Aboualsoltani et al. 2024; Bani et al. 2014; Behmanesh et al. 2019; Direkvand‐Moghadam and Khosravi 2012; Falahieh et al. 2019; Golkhatmy et al. 2024; Heidarifar et al. 2014; Jaafarpour et al. 2015; Jafari et al. 2019; Nahid et al. 2009; Rad et al. 2018; Shabani et al. 2022; Doubova et al. 2007; Leemud et al. 2024; Sriyakul et al. 2012; Zali et al. 2023), while 21 RCTs included a placebo control group (Jahangirifar et al. 2018; Chai et al. 2020; Agarwal and Chaudhary 2023; Aboualsoltani et al. 2024; Atallahi et al. 2014; Behmanesh et al. 2019; Heidarifar et al. 2014; Hesami et al. 2021; Jaafarpour et al. 2015; Jenabi 2013; Fletcher et al. 2013; Doubova et al. 2007; Amanak 2020). The most frequently used instrument for pain assessment was the VAS, reported in 42 studies (Jahangirifar et al. 2018; Abadian et al. 2016; Dehkordi et al. 2019; Mirabi et al. 2011, 2017; Bokaie et al. 2013; Masoumi et al. 2016; Chai et al. 2020; Abd‐El‐Maeboud et al. 2014; Anjum and Sultana 2019; Banu et al. 2025; Atallahi et al. 2014; Bani et al. 2014; Behmanesh et al. 2019; Direkvand‐Moghadam and Khosravi 2012; Falahieh et al. 2019; Golkhatmy et al. 2024; Heidarifar et al. 2014; Hesami et al. 2021; Jaafarpour et al. 2015; Jafari et al. 2019; Jenabi 2013; Jenabi and Fereidoony 2015; Kashefi et al. 2014; Fletcher et al. 2013; Doubova et al. 2007; Leemud et al. 2024; Amanak 2020).

**TABLE 1:** Summary of randomized clinical trials of herbal medicine or primary dysmenorrhea.

First author year, country	Design/sample size/severity/duration	Age	Intervention	Control	Measurement tools	Primary outcome (pain intensity)	Primary outcome (pain duration)	Secondary outcomes	Side effects
Abadian 2016, Iran (Abadian et al. 2016)	Triple‐blind, placebo‐controlled 70 Moderate, severe 2 cycles	*Teucrium polium* 21 ± 1.91 Mefenamic acid 21 ± 1.18	*Teucrium polium* 250 mg q6h 3 days (*n* = 35)	Mefenamic acid 250 mg (*n* = 35)	VAS 0–10	*Teucrium polium* 3.43 ± 2.3 Mefenamic acid 2.7 ± 1.4 n.s.			n.r.
Abd‐El‐Maeboud 2014, Egypt (Abd‐El‐Maeboud et al. 2014)	Crossover 60 Moderate, severe 120 h		Uzara (*Xysmalobium undulatum*) 80 mg/8 h for 2 doses, then 40 mg/8 h (*n* = 60)	Ibuprofen 400 mg q6h (*n* = 60)	VAS 0–10	Uzara 1.63 ± 0.86 Ibuprofen 1.61 ± 0.83 n.s.		Global evaluation: uzara 48.3% vs. ibuprofen 51.7%, n.s. School absences: uzara 11.7% vs. ibuprofen 13.3%, n.s. Rescue drug: uzara 81.7% vs. ibuprofen 90.0%, n.s.	Uzara 0% Ibuprofen 8.3% *p* < 0.05
Aboualsoltani 2024, Iran (Aboualsoltani et al. 2024)	Double‐blind 105 Moderate, severe 2 cycles	*Citrus aurantium* L. 17.17 ± 1.69 Mefenamic acid 17.33 ± 1.51 Placebo 16.90 ± 1.44	*Citrus aurantium* L. flower extract 250 mg q8h first 3 days (*n* = 35)	Placebo (*n* = 35) Mefenamic acid 250 mg (*n* = 35)	McGill pain ruler	Mefenamic acid 0.80 ± 0.35 *Citrus aurantium* L. 0.68 ± 0.17 Placebo 2.21 ± 0.50 *p* < 0.001	Mefenamic acid 5.15 ± 0.87 *Citrus aurantium* L. 5.20 ± 0.80 Placebo 6.22 ± 1.02 n.s.		*C. aurantium* : drowsiness Mefenamic acid: pyrosis, constipation Placebo: headache
Agarwal 2023, India (Agarwal and Chaudhary 2023)	Double‐blind, placebo‐controlled 60 Moderate, severe 6 h	Turmeric–boswellia–sesame formulation 26.67 ± 4.26 Placebo 26.40 ± 4.55	Turmeric–boswellia–sesame formulation 1000 mg (*n* = 30)	Placebo 1000 mg (*n* = 30)	NRS 11‐point scale, TOTPAR	Pain relief: turmeric–boswellia–sesame formulation 18.9 ± 0.56 Placebo 1.5 ± 0.39 *p* < 0.001			n.r.
Amanak 2020, Turkey (Amanak 2020)	Placebo‐controlled 115 n.r. 3 cycles	Dried fig 21.06 ± 2.01 Cinnamon 21.89 ± 2.14 Placebo 21.56 ± 2.85	Dry figs 2 units tid (*n* = 34)	Cinnamon 420 mg tid (*n* = 33) Placebo (*n* = 31)	VAS 0–10, PDAF	Dried figs 3.2 ± 2.6 Cinnamon 5.4 ± 2.7 Placebo 6.3 ± 0.5 *p* = 0.000	Dried fig 6.5 ± 0.8 min Cinnamon 9.3 ± 0.3 min Placebo13.7 ± 2.6 min *p* = 0.000	MDQ *p* = 0.000 PSS *p* = 0.000 WHOQOL‐BREF‐TR *p* = 0.000	n.r.
Anjum 2019, India (Anjum and Sultana 2019)	Single‐blind, parallel 64 n.r. 2 cycles	*Cassia fistula* Linn pod's pericarp + *Myristica fragrans* Houtt arils decoction + jaggery 24.06 ± 5.95 Placebo 22.72 ± 4.64	*Cassia fistula* Linn pod's pericarp + *Myristica fragrans* Houtt arils decoction + jaggery 180 mL 3 days (*n* = 31)	Mefenamic acid 500 mg (*n* = 33)	VAS 0–10	No pain: 96.77% treatment vs. 81.81% control, n.s.	*Cassia fistula* Linn pod's pericarp + *Myristica fragrans* Houtt arils decoction + jaggery 0.83 ± 4.31 Mefenamic acid 1.03 ± 4.19 n.s.	SF‐12 *p* = 0.0001	n.r.
Atallahi 2014, Iran (Atallahi et al. 2014)	Triple‐blinded 80 Mild, moderate, severe 2 cycles	Wheat germ extract 33.45 ± 5.89 Placebo 32.84 ± 5.58	Wheat germ extract 400 mg 3 capsules QD 17 days (*n* = 42)	Placebo (*n* = 38)	VAS 0–10	P50 (25–75): wheat germ extract 0.606 (0–1.553) Placebo 3.610 (0.0–0.666) *p* = 0.003		Fatigue, headache, mood swings were better in wheat germ (*p* < 0.05)	n.r.
Bani 2014, Iran (Bani et al. 2014)	Double‐blind, cross‐over 92 Moderate, severe 2 cycles	*Rosa damascena* 22.20 ± 2.11 Mefenamic Acid 22.13 ± 2.06	*Rosa damascena* extract 200 mg q6h 3 days (*n* = 46)	Mefenamic acid 250 mg (*n* = 46)	VAS 0–10	No pain in both groups at the third day of second cycle			n.r.
Banu 2025, India (Banu et al. 2025)	Double‐blind 62 Moderate, severe 2 cycles	*Juniperus communis* L. 23.90 ± 5.08 Mefenamic acid 24.35 ± 4.90	*Juniperus communis* L. 750 mg tid 3 days (*n* = 31)	Mefenamic acid 250 mg tid 3 days (*n* = 31)	VAS 0–10, NRS	*Juniperus communis* L. 0.26 ± 0.57 Mefenamic acid 0.06 ± 0.25 n.s.			n.r.
Behmanesh 2019, Iran (Behmanesh et al. 2019)	Double‐blind, placebo‐controlled 169 Moderate, severe 2 cycles	19.5 ± 5.0	*Eryngium caucasicum Trautv* 5 mL tid 5 days (*n* = 48)	Ibuprofen 200 mg tid (*n* = 46) Placebo (*n* = 43)	VAS 0–10, VMS	VAS: *Eryngium caucasicum* Trautv: mild 72.3%, moderate 23.4%, severe 4.2% Ibuprofen: mild 54.3%, moderate 45.7%, severe 0.0% Placebo: mild 2.3%, moderate 55.8%, severe 41.9% *p* < 0.0001 VMS: *Eryngium caucasicum* Trautv 1.0 ± 0.78 Ibuprofen 0.76 ± 0.6 Placebo 2.23 ± 0.75 *p* < 0.0001		Satisfaction *p* = 0.0001	Gastric reflux, nausea, vomiting, menorrhagia
Bokaie 2013, Iran (Bokaie et al. 2013)	Parallel 60 moderate, severe 2 cycles	Fennel 21.07 ± 1.8 Mefenamic acid 21.17 ± 1.6	Fennel 2% 25 drops q6h (*n* = 29)	Mefenamic acid 250 mg q6h (*n* = 30)	VAS 0–10	Fennel 1.7 ± 2.9 Mefenamic acid 1.3 ± 1.3 n.s.		Bleeding severity n.s.	Nausea
Chai 2020, China (Chai et al. 2020)	Double‐blinded, placebo‐controlled 30 Moderate, severe 3 cycles	GeGen decoction 24.73 ± 0.38 Placebo 24.23 ± 0.46	GeGen decoction 150 mL bid 7 days (*n* = 15)	Placebo 150 mL bid 7 days (*n* = 13)	VAS 0–100	GeGen decoction 13.34 ± 3.55 Placebo 43.89 ± 3.90 *p* < 0.001		menstrual fluid n.s.	n.r.
Dehkordi 2019, Iran (Dehkordi et al. 2019)	Double‐blind, crossover 96 Moderate, severe 4 cycles	Salix extract 20.48 ± 1.64 Mefenamic acid 21 ± 1.85	Salix extract 200 mg bid (*n* = 48)	Mefenamic acid 250 mg tid (*n* = 48)	VAS 0–10	Mean difference (SE) 1.61 (0.06) *p* < 0.001		Bleeding n.s.	n.r.
Direkvand‐Moghadam 2012, Iran (Direkvand‐Moghadam and Khosravi 2012)	Single‐blind 120 Mild, moderate, severe 2 cycles		Shirazi *Thymus Vulgaris* 5 ml QID (*n* = 60)	Ibuprofen 400 mg (*n* = 60)	VAS 0–10	n.s.	n.s.		n.r.
Doubova 2007, Mexico (Doubova et al. 2007)	Double‐blinded 197 Moderate, severe 4 cycles	Phyto‐drug 3 mg 19.6 ± 2.0 Phyto‐drug 6 mg 19.4 ± 1.7 Placebo 19.5 ± 1.9 Ibuprofen 19.9 ± 2.2	*Psidium guajava* folium extract 3 mg/day 5 days (*n* = 52) *Psidium guajava* folium extract 6 mg/day 5 days (*n* = 57)	Ibuprofen 1200 mg/day 5 days (*n* = 46) Placebo 3 mg/day 5 days (*n* = 42)	VAS 0–10	Phyto‐drug 3 mg 3.39 ± 2.18 Phyto‐drug 6 mg 4.31 ± 2.50 Placebo 3.18 ± 2.08 Ibuprofen 3.22 ± 2.05 n.s.			Abdominal pain, nausea
Falahieh 2019, Iran (Falahieh et al. 2019)	Double‐blind, placebo‐controlled 70 Moderate, severe 2 cycles	*Nigella sativa* 22.9 ± 3.54 Mefenamic acid 22.14 ± 3.02	*Nigella sativa* 1 g q8h 3 days (*n* = 35)	Mefenamic acid 250 mg q8h 3 days (*n* = 35)	VAS 0–10	*Nigella sativa* 1.50 ± 0.98 Mefenamic Acid 1.91 ± 1.02 *p* = 0.045	*Nigella sativa* 1.45 ± 0.45 Mefenamic Acid 1.46 ± 0.45 *p* = 0.001	Fatigue *p* = 0.001 mood swings *p* = 0.001 nausea, vomiting, lack of energy, headache, diarrhea, syncope n.s.	n.r.
Fletcher 2013 Jamaica (Fletcher et al. 2013)	Double‐blind, placebo‐controlled 100 n.r. 3 cycles	*Morinda citrifolia* 22.7 ± 5.4 Placebo 22 ± 3.9	*Morinda citrifolia* 400 mg bid 5 days (*n* = 42)	Placebo bid 5 days (*n* = 38)	VAS 0–10	*Morinda citrifolia* 7.0 ± 2.5 Placebo 6.0 ± 3.1 n.s.			n.r.
Golkhatmy 2024, Iran (Golkhatmy et al. 2024)	Double‐blind 82 Moderate, severe 2 cycles	Rosemary group 21.64 ± 0.26 Mefenamic acid 22.32 ± 0.31	Rosemary 250 g q8h 3 days (*n* = 42)	Mefenamic Acid q8h 3 days (*n* = 40)	VAS 0–10, SF‐36	Rosemary group 2.35 ± 1.27 Mefenamic acid 2.82 ± 1.72 *p* = 0.07		SF‐36 n.s.	n.r.
Heidarifar 2014, Iran (Heidarifar et al. 2014)	Double‐blind 75 Moderate, severe 2 cycles	*Anethum graveolens* 20.95 ± 1.82 Mefenamic acid 22.04 ± 3.07 Placebo 20.95 ± 1.60	*Anethum graveolens* 1000 mg q12h 5 days (*n* = 23)	Mefenamic acid 250 mg q12h (*n* = 24) Placebo 500 mg q12h 5 days (*n* = 23)	VAS 0–10, VMS	*Anethum graveolens* 4.13 ± 1.76 Mefenamic acid 3.78 ± 1.4 Placebo 5.08 ± 1.44 *p* = 0.030		VMS: *Anethum graveolens* : 14.3% mild, 11.4% moderate, 1.4% severe Mefenamic acid: 10% mild, 14.3% moderate, 0% severe Placebo: 14.3% mild, 4.3% moderate, 0% severe	Menstrual changes, gastrointestinal discomfort
Hesami 2021, Iran (Hesami et al. 2021)	Double‐blind, placebo‐controlled 121 Mild, moderate, severe 2 cycles	Turmeric 22.11 ± 2.09 Mefenamic acid 23.01 ± 3.02 Turmeric + mefenamic acid 22.37 ± 2.41 Placebo 23.19 ± 1.99	Turmeric 500 mg 5 days (*n* = 30) Mefenamic acid 250 mg 5 days (*n* = 30) Turmeric 500 mg + Mefenamic acid 250 mg 5 days (*n* = 30)	Placebo 5 days (*n* = 31)	VAS 0–10	Turmeric 5.67 ± 0.8 Mefenamic acid 6.14 ± 0.19 Turmeric + mefenamic acid 4.86 ± 0.1 Placebo 7.01 ± 0.28 *p* < 0.05			n.r.
Jaafarpour 2015, Iran (Jaafarpour et al. 2015)	Double‐blind 114 moderate 72 h	Cinnamon 20.7 ± 1.1 Ibuprofen 20.8 ± 1.1 Placebo 21.3 ± 1.5	Cinnamon 420 mg tid (*n* = 38)	Ibuprofen 400 mg tid (*n* = 38) Placebo tid (*n* = 38)	VAS 0–10	Cinnamon 1.8 ± 0.4 Ibuprofen 0.6 ± 0.6 Placebo 4.0 ± 0.3 *p* < 0.001	Cinnamon 3.2 ± 0.4 Ibuprofen 0.6 ± 0.4 Placebo 18.7 ± 1.3 *p* < 0.001		n.r.
Jafari 2019, Iran (Jafari et al. 2019)	Triple‐blind 60 Moderate, severe 2 cycles	*Glycyrrhiza glabra* 22.73 ± 1.91 Ibuprofen 22.46 ± 1.79	*Glycyrrhiza glabra* L. 5 cc bid 5 days (*n* = 26)	Ibuprofen 400 mg q8h 5 days (*n* = 24)	VAS 0–10	*G. glabra* 0.31 ± 0.84 Ibuprofen 0.08 ± 0.28 n.s.			Ibuprofen: heartburn, stomachache
Jahangirifar 2018, Iran (Jahangirifar et al. 2018)	Double blind 80 Moderate, severe 2 cycles	Cinnamon 22.2 ± 2.2 Placebo 22.3 ± 2.7	Cinnamon 1000 mg tid 3 days (*n* = 30)	Placebo 1000 mg tid 3 days (*n* = 28)	VAS 0–10	Cinnamon 3.2 ± 2.4 Placebo 4.9 ± 2.1 *p* = 0.002			n.r.
Jenabi 2013, Iran (Jenabi 2013)	Parallel 70 Moderate, severe 1 cycle	Ginger 21.33 ± 1.16 Placebo 21.54 ± 1.78	Ginger 500 mg tid 3 days (*n* = 35)	Placebo 3 days (*n* = 34)	VAS 0–10	Ginger 4.81 ± 1.70 Placebo 7.11 ± 1.12 *p* = 0.001			n.r.
Jenabi 2015, Iran (Jenabi and Fereidoony 2015)	Double‐blind 96 Moderate, severe 2 cycles	*Achillea millefolium* 21.66 ± 5.77 Placebo 20.37 ± 6.0	*Achillea millefolium* 4 g tid 3 days (*n* = 45)	Placebo 4 g tid 3 days (*n* = 46)	VAS 0–10	Baseline minus post therapy: *Achillea millefolium* 1.59 ± 1.81 Placebo 0.41 ± 0.79 *p* < 0.0001			n.r.
Kashefi 2014, Iran (Kashefi et al. 2014)	Placebo‐Controlled 150 Moderate, severe 2 cycles	range, 14–18 years	Ginger 240 mg tid 4 days (*n* = 48)	Zinc sulfate 220 mg tid 4 days (*n* = 56) Placebo tid 4 days (*n* = 46)	VAS 0–10	Ginger 3.08 ± 1.52 Zinc sulfate 3.12 ± 1.2 Placebo 6.95 ± 1.67 *p* < 0.001			Diarrhea, headache, heartburn
Leemud 2024, Thailand (Leemud et al. 2024)	Single‐blind 74 Mild, moderate 3 cycles	Leard‐Ngam 19.91 ± 1.14 Mefenamic acid 19.97 ± 1.28	Leard‐Ngam formula 500 mg 2 capsules tid 3 days (*n* = 35)	Mefenamic acid 250 mg 2 capsules tid 3 days (*n* = 37)	VAS 0–10, VMS	Leard‐Ngam 3.23 ± 1.89 Mefenamic acid 3.03 ± 1.70 n.s.		VMS: Leard‐Ngam 1.03 ± 0.70 Mefenamic acid 0.97 ± 0.68 n.s.	Leard‐Ngam: dizziness, nausea, vomiting Mefenamic acid: nausea, vomiting, severe abdominal pain, decrease in bleeding, heavy periods
Masoumi 2016, Iran (Masoumi et al. 2016)	Double‐blinded, crossover 126 n.r. 2 cycles	20.99 ± 0.15	Peppermint oil capsule QD 3 days (*n* = 63)	Mefenamic acid 250 mg q8h 3 days (*n* = 63)	VAS 0–10, PBAC	Peppermint 3.12 ± 1.87 Mefenamic acid 2.82 ± 2.00 *p* = 0.098	Peppermint 1.07 ± 1.06 Mefenamic acid 0.87 ± 0.91 *p* = 0.017	Bleeding: Peppermint 41.32 ± 26.02 Mefenamic acid 36.57 ± 23.47 *p* = 0.001 Peppermint: less nausea, vomiting, diarrhea, analgesic usage	n.r.
Mirabi 2011, Iran (Mirabi et al. 2011)	Double‐blind, placebo‐controlled 100 Moderate, severe 2 cycles	Valerian 20.90 ± 1.36 Placebo 21.04 ± 1.54	Valerian 255 mg tid 3 days (*n* = 51)	Placebo tid 3 days (*n* = 49)	VAS 0–10	Valerian 1.99 ± 1.43 Placebo 4.41 ± 1.76 *p* < 0.001		Syncope: Valerian 0.10 ± 0.45 Placebo 0.31 ± 0.65 *p* = 0.006	n.r.
Mirabi 2017, Iran (Mirabi et al. 2017)	Double‐blind 110 Moderate, severe 2 cycles	*Melissa Officinalis* 21.08 ± 1.34 Placebo 21.14 ± 1.61	*Melissa Officinalis* 330 mg tid 3 days (*n* = 55)	Placebo 330 mg tid 3 days (*n* = 55)	VAS 0–10	*Melissa Officinalis* 2.00 ± 1.43 Placebo 3.03 ± 1.61 *p* < 0.001			n.r.
Modaress Nejad 2006, Iran (Modaress Nejad and Asadipour 2006)	Double‐blind 120 Moderate, severe 2 cycles	Fennel 15.5 ± 1.5 Mefenamic acid 15.5 ± 1.4	Fennel extract 30 drops q6h 3 days (*n* = 55)	Mefenamic acid 250 mg q6h 3 days (*n* = 55)	VMSS	Fennel: 80% no pain, 18% moderate, 2% severe Mefenamic acid: 73% no pain, 20% moderate, 7% severe n.s.			n.r.
Nahid 2009, Iran (Nahid et al. 2009)	Double‐blind, placebo‐controlled 180 Mild, moderate, severe 3 cycles	Herbal drug 20.6 ± 3.2 Mefenamic acid 20.5 ± 3.3 Placebo 20 ± 3	Herbal drug (saffron, celery seed, anise) 500 mg tid 3 days (*n* = 57)	Mefenamic acid 250 mg tid 3 days (*n* = 55) Placebo tid 3 days (*n* = 51)	VAS 0–10	Herbal: 35% no pain, 36.8% mild, 21% moderate, 7% severe Mefenamic acid: 18% no pain, 41.8% mild, 29% moderate, 11% severe Placebo: 3.9% no pain, 29.4% mild, 55% moderate, 9.8% severe *p* < 0.01		Rescue medication: Herbal 18%, Mefenamic acid 20%, Placebo 60%	n.r.
Nuha 2023, Indonesia (Nuha et al. 2023)	Single‐blind 45 Mild, moderate, severe 24 h	range, 17–21 years	Dark chocolate 70% 35 g (*n* = 15) Coconut Water 330 mL (*n* = 15)	Ibuprofen 400 mg (*n* = 15)	NRS	Dark chocolate: 33.3% no pain, 46.7% mild, 20% moderate Coconut Water: 0% no pain, 73.3% mild, 26.7% moderate Ibuprofen: 66.7% no pain, 26.7% mild, 6.7% moderate *p* = 0.004			n.r.
Rad 2018, Iran (Rad et al. 2018)	Crossover 168 Moderate, severe 2 cycles	Ginger 22.35 ± 2.10 Novafen 21.43 ± 2.28	Ginger 200 mg q6h (*n* = 78)	Novafen 200 mg q6h (*n* = 90)	VAS 0–10, MVRS, PBAC	Ginger 2.97 ± 2.69 Novafen 3.10 ± 2.69 n.s.		bleeding n.s.	n.r.
Rahnama 2012, Iran (Rahnama et al. 2012)	Double‐blind, placebo‐controlled, Parallel 118 Moderate, severe 2 cycles	Ginger 21.4 ± 2.0 Placebo 21.3 ± 2.2	Ginger 500 mg tid 5 days (*n* = 59)	Placebo 500 mg tid 5 days (*n* = 46)	VAS 0–10, VMS	Ginger 4.61 ± 2.55 Placebo 6.01 ± 2.65 *p* = 0.029	Ginger 10.88 ± 14.54 Placebo 15.57 ± 14.72 *p* = 0.210		Ginger: heatburn Placebo: nausea
Rehman 2015, India (Rehman et al. 2015)	Single‐blind 45 Moderate, severe 3 cycles	Rhubarb 18.87 ± 2.47 Mefenamic acid 17.73 ± 1.94	Rhubarb 500 mg 3 capsules bid 5 days (*n* = 30)	Mefenamic acid 250 mg tid (*n* = 15)	VAS 0–10, VMSS, QOL	Rhubarb 2.77 ± 0.29 Mefenamic acid 2.27 ± 0.46 *p* = 0.094	Rhubarb 1.10 ± 0.10 Mefenamic acid 0.87 ± 0.19 *p* = 0.242	VMSS: Rhubarb 0.93 ± 0.08 Mefenamic acid 0.87 ± 0.13 *p* = 0.642 QOL n.s.	Rhubarb: bloating, diarrhoea
Rezaeyan 2015 Iran (Rezaeyan et al. 2015)	Single‐blinded, crossover 60 Moderate, severe 2 cycles + 1 cycle wash out + 2 cycles	Extra virgin olive oil 21.86 ± 2.35 Ibuprofen 22.86 ± 2.35	Extra virgin olive oil 25 cc QD 14 days (*n* = 30)	Ibuprofen 400 mg tid 3 days (*n* = 30)	VAS 0–10	Extra virgin olive oil 1.1 ± 0.8 Ibuprofen 3.8 ± 2.2 *p* = 0.001			n.r.
Safdari‐Dehcheshmehi 2016, Iran (Safdari‐Dehcheshmehi and Parvin 2016)	Single‐blind 43 Moderate, severe 3 cycles	*Melissa officinalis* 24.55 ± 4.78 Mefenamic acid 25.38 ± 7.71	*Melissa officinalis* 1 tea bag q8h (*n* = 22)	Mefenamic Acid 250 mg q8h 3 days (*n* = 21)	VAS 0–10	*Melissa officinalis* 3.16 ± 0.63 Mefenamic Acid 4.09 ± 1.70 *p* = 0.008	*Melissa officinalis* 24.77 ± 12.46 Mefenamic acid 30.23 ± 50.01 *p* = 0.101		n.r.
Sardashti 2020, Iran (Sardashti et al. 2020)	Single‐blind 150 n.r. 2 cycles	*Aloe vera* gel 22.1 ± 1.4 Ibuprofen 20.4 ± 1.2	*Aloe vera* gel pills 10 mg QID (*n* = 43)	Ibuprofen tid (*n* = 37)	VMS	*Aloe vera* gel 2.02 ± 0.34 Ibuprofen 1.32 ± 0.15 *p* = 0.61	n.s.		n.r.
Shabani 2022, Iran (Shabani et al. 2022)	Double‐blind 200 Moderate, severe 2 cycles	Chamomile 21.5 ± 2.24 Mefenamic acid 20.88 ± 1.74	Chamomile 5000 mg + 1 spoon honey tid 5 days (*n* = 95)	Mefenamic Acid 250 mg tid 5 days (*n* = 96)	VAS 0–10, PBAC, VMSS	Chamomile: 10.4% no pain, 45.8% mild, 37.5% moderate, 6.3% severe Mefenamic acid: 4.2% no pain, 53% mild, 36.5% moderate, 6.3% severe *p* = 0.332	Chamomile 1.82 ± 0.57 Mefenamic acid 1.70 ± 0.73 *p* = 0.300	PBAC *p* = 0.131	n.r.
Shirvani 2015, Iran (Shirvani et al. 2015)	Parallel 122 Moderate, severe 2 cycles	Ginger 21.60 ± 2.14 Mefenamic acid 21.62 ± 2.0	Ginger 250 mg q6h (*n* = 60)	Mefenamic acid 250 mg q8h (*n* = 60)	VAS 0–100	Ginger 38.19 ± 20.47 Mefenamic acid 33.75 ± 17.71 n.s.			n.r.
Sriyakul 2012, Thailand (Sriyakul et al. 2012)	Double‐blind 207 Moderate, severe 6 cycles	Prasaplai 19.78 + 1.62 Mefenamic acid 19.73 + 1.52	Prasaplai Herbal Extract 200 mg 2 pills tid 3 days (*n* = 103)	Mefenamic acid 250 mg 2 pills tid 3 days (*n* = 104)	VAS 0–10	Prasaplai 2.85 ± 0.22 Mefenamic acid 3.27 ± 0.22 n.s.			n.r.
Tabari 2020, Iran (Tabari et al. 2020)	Double blind 74 Moderate, severe 2 cycles		Turmeric 500 mg 2 pills 3 days (*n* = 34)	Placebo 10 mg (*n* = 34)	VAS 0–10, VMSS	Curcumin 4.6 ± 1.5 Placebo 5.8 ± 1.82 *p* = 0.004	Curcumin 9.9 ± 18.26 Placebo 16.9 ± 11.85 *p* < 0.001		n.r.
Vannabhum 2016, Thailand (Vannabhum et al. 2016)	Double‐blind, placebo‐controlled 40 Moderate, severe 3 days	Prasaplai 22.5 ± 4.2 Placebo 26.6 ± 7.5	Prasaplai formula 500 mg 2 pills tid 3 days (*n* = 20)	Placebo 500 mg 2 pills tid 3 days (*n* = 20)	NRS	n.s.			n.r.
Younesy 2014, Iran (Younesy et al. 2014)	Double‐blind, placebo‐controlled 106 Moderate, severe 2 cycles	Fenugreek 19.86 ± 1.52 Placebo 20 ± 1.56	Fenugreek seed 900 mg TID 3 days (*n* = 51)	Placebo (*n* = 50)	VAS 0–10	Fenugreek 3.25 ± 1.25 Placebo 5.96 ± 1.87 *p* < 0.001		Fenugreek: less use of sedative tablets and symptoms associated to dysmenorrhea	n.r.
Zali 2023, Iran (Zali et al. 2023)	Parallel 70 Moderate, severe 3 cycles	Ajwain 25.80 ± 5.69 Mefenamic acid 25.14 ± 5.79	Ajwain ( *Trachyspermum ammi* L.) 500 mg tid 7 days (*n* = 26)	Mefenamic Acid 250 mg q8h (*n* = 27)	VAS 0–10, VMSS	Ajwain 6.00 ± 2.62 Mefenamic acid 6.03 ± 1.97 *p* = 0.96	Ajwain 10.23 ± 10.71 Mefenamic acid 7.92 ± 6.95 *p* = 0.36		Ajwain: acne, constipation, spotting Mefenamic acid: stomach pain, constipation, bloating
Zeraati 2014, Iran (Zeraati et al. 2014)	Double‐blind 105 Mild, moderate 2 cycles	Fennelin 21.60 ± 2.59 Vitagnus 20.88 ± 1.23 Mefenamic acid 22.20 ± 3.72 Placebo 21.60 ± 2.59	Fennelin 30 drops q4h 4 days (*n* = 25) Vitagnus 40 drops QD 4 days (*n* = 25)	Mefenamic acid 250 mg q4h 4 days (*n* = 30) Placebo 30 drops q4h 4 days (*n* = 25)	VAS 0–10	Fennelin 1.3 ± 2.2 Vitagnus 1.7 ± 3.2 Mefenamic acid 1.7 ± 3.6 Placebo 1.3 ± 4.1 n.s.			n.r.

Abbreviations: bid, Twice a day; MVRS, Multidimensional Verbal Rating Scale; NRS, Numerical Rating Scale; n.r., not reported; n.s., not significant; PBAC, Pictorial Blood Assessment Chart; PDAF, Pain Duration Assessment Form; QD, Once a day; QID, Four times a day; QOL, Quality of Life; SF‐***12***, Short Form‐12 Health Survey; SF‐36, Short Form‐36 Health Survey; tid, three times a day; TOTPAR, Total Pain Relief; VAS, Visual Analogue Scale; VMSS, Verbal Multidimensional Scoring System; WHOQOL‐BREF‐TR, World Health Organization Quality of Life.

### 3.2 RoB 2.0

**FIGURE 2:**
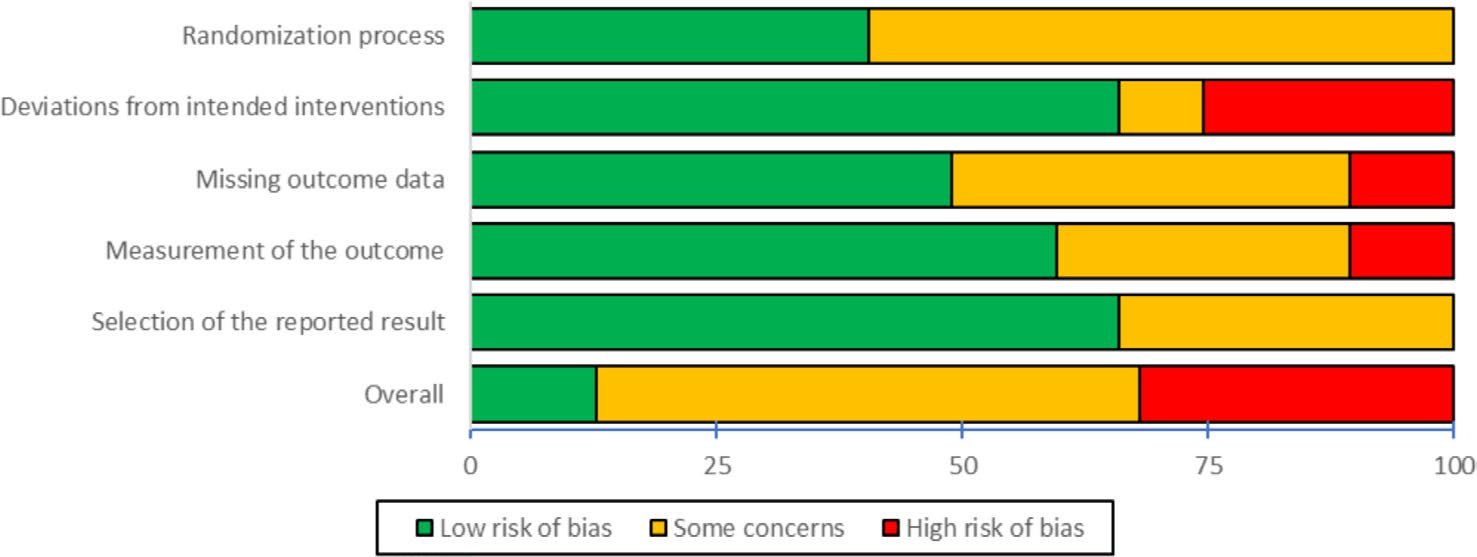
Risk of bias graph: Review authors' judgments about each risk of bias presented as percentages across all included studies.

**FIGURE 3:**
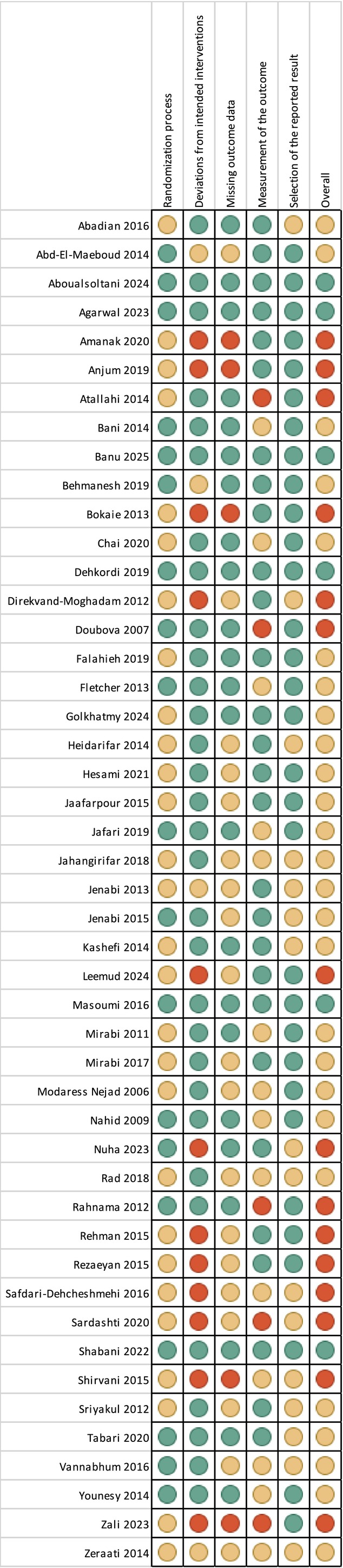
Risk of bias summary: Review authors' judgments about each risk of bias item for each included study.

The overall RoB was predominantly rated as “some concerns,” with nearly one‐third of the trials judged at high risk. Issues were most evident in the randomization process and incomplete outcome data, whereas selective reporting was less problematic (Figures 2 and 3).

### 3.3 Outcome Measurements

**FIGURE 4:**
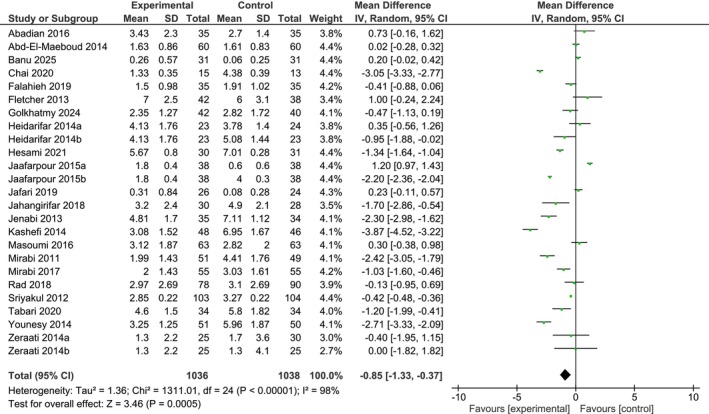
Forest plot of effects of plant‐based treatment on primary dysmenorrhea compared with control group (VAS scores standardized to 0–10).

In this meta‐analysis, 22 studies met the inclusion criteria (Jahangirifar et al. 2018; Abadian et al. 2016; Mirabi et al. 2011, 2017; Masoumi et al. 2016; Zeraati et al. 2014; Chai et al. 2020; Abd‐El‐Maeboud et al. 2014; Banu et al. 2025; Falahieh et al. 2019; Golkhatmy et al. 2024; Heidarifar et al. 2014; Hesami et al. 2021; Jaafarpour et al. 2015; Jafari et al. 2019; Jenabi 2013; Kashefi et al. 2014; Rad et al. 2018; Tabari et al. 2020; Fletcher et al. 2013; Sriyakul et al. 2012; Younesy et al. 2014). The results showed the effects of plant‐based treatment on primary dysmenorrhea compared to the control group (*n* = 2074, MD: −0.85, 95% CI: −1.33 to −0.37, *p* = 0.0005, *I* ^2^ = 98%; Figure 4).

**FIGURE 5:**
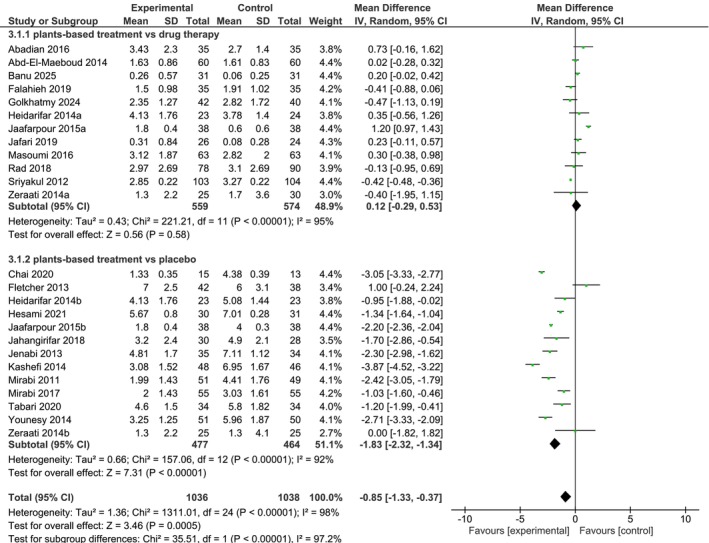
Forest plot of effects of plant‐based treatment on primary dysmenorrhea compared with drug therapy and placebo (VAS scores standardized to 0–10).

**FIGURE 6:**
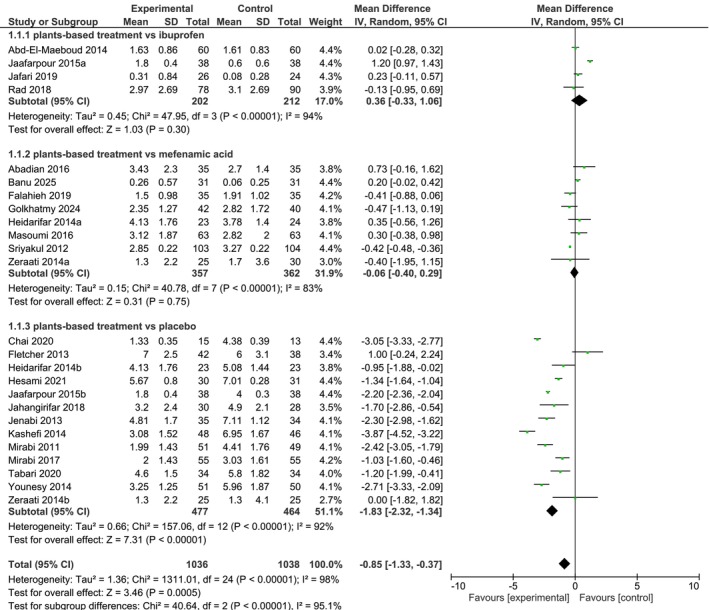
Forest plot of effects of plant‐based treatment on primary dysmenorrhea compared with ibuprofen, mefenamic acid, and placebo (VAS scores standardized to 0–10).

Due to the high heterogeneity, a subgroup analysis was performed based on the type of control (placebo or drug therapy; Figure 5). When comparing plant‐based treatment with drug therapy, no statistically significant differences were observed (*n* = 1133, MD: 0.12, 95% CI: −0.29 to 0.53, *p* = 0.58, *I* ^2^ = 95%), suggesting comparable efficacy between the two interventions. In contrast, when plant‐based treatment was compared with placebo, the plant‐based treatment was found to be significantly more effective (*n* = 941, MD: −1.83, 95% CI: −2.32 to −1.34, *p* < 0.00001, *I* ^2^ = 92%). Subsequently, an additional analysis was conducted considering specific control types (placebo, mefenamic acid, or ibuprofen; Figure 6). No statistically significant differences were found between plant‐based treatment and ibuprofen (*n* = 414, MD: 0.36, 95% CI: −0.33 to 1.06, *p* = 0.30, *I* ^2^ = 94%) or mefenamic acid (*n* = 719, MD: −0.06, 95% CI: −0.40 to 0.29, *p* = 0.75, *I* ^2^ = 83%), suggesting similar efficacy across these interventions.

### 3.4 Publication Bias and Heterogeneity

The funnel plot analysis revealed asymmetry in the MD estimate of pain intensity, indicating a potential publication bias (Figure S1), likely due to the small sample sizes of the studies and the high variability in intervention types. The leave‐one‐out analysis revealed that heterogeneity remained very high (*I* ^2^ > 90%) after sequentially excluding each individual study (Table S1), indicating that no single study accounted for the observed heterogeneity.

### 3.5 Adverse Effects

Twelve studies reported adverse events in the intervention and/or control groups. However, these events were mild and resolved upon discontinuation of the studied product. The most frequently reported adverse event was nausea, documented in multiple studies (Rahnama et al. 2012; Bokaie et al. 2013; Abd‐El‐Maeboud et al. 2014; Rehman et al. 2015; Aboualsoltani et al. 2024; Behmanesh et al. 2019; Heidarifar et al. 2014; Jafari et al. 2019; Kashefi et al. 2014; Doubova et al. 2007; Leemud et al. 2024; Zali et al. 2023).

### 3.6 Assessment of Evidence Quality and Recommendation Level

The quality of the evidence and the level of recommendation, as assessed by the GRADE system, was rated as “very low” (Data S1). This rating is primarily attributed to the high heterogeneity of the treatment types employed, small sample sizes, and a moderate risk of bias.

## 4 Discussion

Forty‐seven RCTs were identified that met the inclusion criteria, evaluating the effect of medicinal plants with analgesic properties in primary dysmenorrhea. The results indicate that, in general, the studied plants show effects comparable to those of conventional drugs used in the treatment of primary dysmenorrhea, such as mefenamic acid or ibuprofen, and are more effective compared to a neutral placebo. Only 12 studies reported adverse effects (Rahnama et al. 2012; Bokaie et al. 2013; Abd‐El‐Maeboud et al. 2014; Rehman et al. 2015; Aboualsoltani et al. 2024; Behmanesh et al. 2019; Heidarifar et al. 2014; Jafari et al. 2019; Kashefi et al. 2014; Doubova et al. 2007; Leemud et al. 2024; Zali et al. 2023), which limits the ability to assess the safety of the treatment in the studies that did not document them. However, the reported adverse effects were classified as mild, occurring sporadically and resolving upon treatment discontinuation.

Other systematic reviews and meta‐analyses have addressed the use of medicinal plants in the treatment of dysmenorrhea. Mirabi et al. (2014) conducted a review focusing on various medicinal plants, covering a global search for relevant species. Their findings were consistent with those of the present review. However, they used more lenient inclusion criteria and quality assessments. (Chehreh et al. (2021)), in their systematic review and meta‐analysis of studies conducted in Iran, compared the effects of medicinal plants with NSAIDs. They found no significant differences between the two treatments, although they noted that prolonged use of medicinal plants may be associated with greater pain relief compared to NSAIDs.

Ginger is one of the most studied plants and is widely used in the Chinese population for menstrual pain relief. Other studies have corroborated that ginger has a superior analgesic effect compared to placebo and shows no significant differences when compared to mefenamic acid, reinforcing its efficacy in the treatment of dysmenorrhea. However, many of these studies present a high risk of bias, which undermines the robustness of the results obtained in their meta‐analysis, in addition to considerable heterogeneity despite all included studies using ginger (Chen et al. 2016; Daily et al. 2015).

A considerable number of studies on the use of fennel have been identified, although several were excluded due to non‐compliance with the established inclusion criteria. Lee et al. (2020) reported that fennel, compared to a placebo, has potential in pain relief, showing effects similar to those of conventional drugs.

In China, the treatment of various ailments with combined medicinal plant formulas is a common practice. Formulas such as Wenjing decoction, Taohong Siwu Tang, Shaofu Zhuyu decoction, Danggui Shaoyao San, Xuefu Zhuyu decoction, Siwu Tang, and Sini decoction have been studied, showing therapeutic potential. However, the quality of the available evidence is low due to a high risk of bias in the included studies (Leem et al. 2019; Gao et al. 2017; Ji et al. 2020; Lee, Choi, et al. 2016; Lee, Jun, et al. 2016; Li et al. 2020; Ma et al. 2021). To improve the quality of evidence in traditional Chinese medicine, it is imperative to enhance the design and execution methods of clinical trials (Wang et al. 2023).

It is important to acknowledge several limitations of our review. Most of the included studies had a relatively short duration of approximately two menstrual cycles, with interventions typically administered during the first three days of each cycle. Longer‐term studies would have been valuable to determine whether the observed benefits are sustained beyond the immediate effects. In addition, the majority of studies were conducted in Iran, and although many studies from other Middle Eastern countries were identified, they could not be included due to language restrictions. The RoB assessment indicated that the overall quality of the included studies was moderate. Despite performing extensive subgroup analyses based on intervention type, timing of administration, dosage, number of treatment cycles, age group, geographic region, and comparator, heterogeneity remained very high (*I* ^2^ > 90%). This substantial heterogeneity, confirmed in RevMan analyses, was largely attributable to small sample sizes and the wide variability in intervention types. While our findings suggest that plant‐based treatments may be more effective than placebo, no significant differences were observed when compared with ibuprofen or mefenamic acid. These results should therefore be interpreted with caution, and future studies should aim to reduce heterogeneity and improve methodological rigor to provide more reliable and clinically meaningful conclusions.

## 5 Conclusions

This systematic review and meta‐analysis underscores the promising potential of plant‐based treatments in alleviating pain associated with primary dysmenorrhea in women. Although the results suggest positive outcomes, this study emphasizes the importance of conducting additional clinical studies with larger sample sizes and rigorous methodologies to validate these findings and establish clearer therapeutic guidelines.

## Author Contributions

**Lifei Wu:** conceptualization, formal analysis, data curation, writing – original draft. **Inmaculada Xu Lou:** conceptualization, methodology, writing – review and editing, supervision. **Zhenting Hu:** formal analysis, data curation, funding acquisition. **Gan Wang:** investigation, data curation, project administration. **Sammit Vishram Deshpande:** investigation, data curation, project administration, funding acquisition. **Rocío Cáceres‐Matos:** methodology, writing – review and editing, supervision. All the authors listed have made substantial, direct, and intellectual contributions to the work and approved it for publication.

## Funding

The authors have nothing to report.

## Ethics Statement

The authors have nothing to report.

## Consent

The authors have nothing to report.

## Conflicts of Interest

The authors declare no conflicts of interest.

## Supporting information


**Figure S1:** Funnel plot for plants‐based treatment for primary dysmenorrhea.
**Table S1:** Leave‐one‐out sensitivity analysis of the meta‐analysis.

**Data S1:** ptr70324‐sup‐0002‐Supplementary_material_2.pdf.

**Data S2:** ptr70324‐sup‐0003‐PRISMA_2020_checklist.docx.

## Data Availability

The data that support the findings of this study are available from the corresponding author upon reasonable request.
