# Supplementary material for: Efficacy of Plant‐Derived Therapies for Primary Dysmenorrhea: A Systematic Review and Meta‐Analysis of Randomized Controlled Trials
Source: Phytother Res. 2026 Apr 15;40(6):3818–39. doi: 10.1002/ptr.70324 (PMC13254016; doi:10.1002/ptr.70324)
Supplement: Supplementary file 1 — Figure S1: Funnel plot for plants‐based treatment for primary dysmenorrhea. Table S1: Leave‐one‐out sensitivity analysis of the meta‐analysis. [file PTR-40-3818-s001.docx]

**
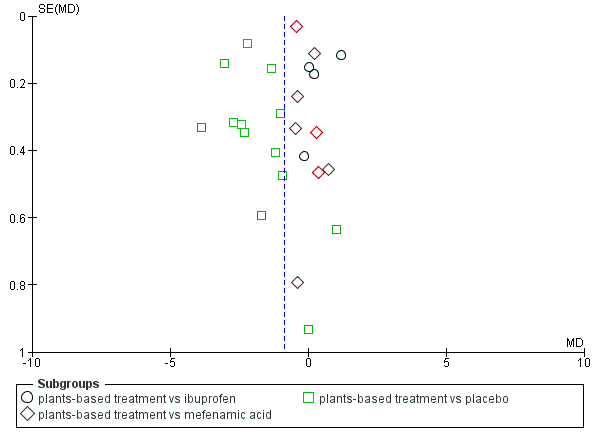
**

**Figure S1.** Funnel plot for plants-based treatment for primary dysmenorrhea

**Table S1.** Leave-one-out sensitivity analysis of the meta-analysis

| Excluded study | MD (95% CI) | P value | I^2^ |
| --- | --- | --- | --- |
| Abadian 2016 | -0.91 [-1.41, -0.42] | <0.00001 | 98% |
| Abd-El-Maeboud 2014 | -0.89 [-1.39, -0.38] | <0.00001 | 98% |
| Banu 2025 | -0.90 [-1.41, -0.39] | <0.00001 | 98% |
| Chai 2020 | -0.75 [-1.20, -0.30] | <0.00001 | 98% |
| Falahieh 2019 | -0.87 [-1.37, -0.37] | <0.00001 | 98% |
| Fletcher 2013 | -0.92 [-1.41, -0.43] | <0.00001 | 98% |
| Golkhatmy 2024 | -0.87 [-1.36, -0.37] | <0.00001 | 98% |
| Heidarifar 2014a | -0.90 [-1.39, -0.41] | <0.00001 | 98% |
| Heidarifar 2014b | -0.85 [-1.34, -0.35] | <0.00001 | 98% |
| Hesami 2021 | -0.83 [-1.33, -0.32] | <0.00001 | 98% |
| Jaafarpour 2015a | -0.95 [-1.42, -0.48] | <0.00001 | 98% |
| Jaafarpour 2015b | -0.79 [-1.25, -0.33] | <0.00001 | 97% |
| Jafari 2019 | -0.90 [-1.40, -0.40] | <0.00001 | 98% |
| Jahangirifar 2018 | -0.82 [-1.31, -0.33] | <0.00001 | 98% |
| Jenabi 2013 | -0.79 [-1.28, -0.30] | <0.00001 | 98% |
| Kashefi 2014 | -0.72 [-1.20, -0.25] | <0.00001 | 98% |
| Masoumi 2016 | -0.90 [-1.39, -0.41] | <0.00001 | 98% |
| Mirabi 2011 | -0.78 [-1.27, -0.29] | <0.00001 | 98% |
| Mirabi 2017 | -0.84 [-1.34, -0.35] | <0.00001 | 98% |
| Rad 2018 | -0.88 [-1.37, -0.39] | <0.00001 | 98% |
| Sriyakul 2012 | -0.86 [-1.48, -0.24] | <0.00001 | 98% |
| Tabari 2020 | -0.84 [-1.33, -0.34] | <0.00001 | 98% |
| Younesy 2014 | -0.77 [-1.26, -0.28] | <0.00001 | 98% |
| Zeraati 2014a | -0.86 [-1.35, -0.38] | <0.00001 | 98% |
| Zeraati 2014b | -0.87 [-1.36, -0.39] | <0.00001 | 98% |
