# Supplementary material for: Efficacy of Plant‐Derived Therapies for Primary Dysmenorrhea: A Systematic Review and Meta‐Analysis of Randomized Controlled Trials
Source: Phytother Res. 2026 Apr 15;40(6):3818–39. doi: 10.1002/ptr.70324 (PMC13254016; doi:10.1002/ptr.70324)
Supplement: Supplementary file 2 — Data S1: ptr70324‐sup‐0002‐Supplementary_material_2.pdf. [file PTR-40-3818-s003.pdf]

Author(s):
Question: Intervention compared to control for primary dysmenorrhea
Setting:
Bibliography:

Certainty assessment							№ of patients		Effect		Certainty	Importance
**№ of studies**	**Study design**	**Risk of bias**	**Inconsistency**	**Indirectness**	**Imprecision**	**Other considerations**	**intervention**	**control**	**Relative (95% CI)**	**Absolute (95% CI)**		
**pain intensity**												
25	randomised trials	serious[a]	very serious[b]	serious[c]	serious[d]	none	1036	1038	-	MD **0.85 lower** (1.33 lower to 0.37 lower)	⊕◯◯◯ Very low[a,b,c,d]	CRITICAL
**pain intensity - plants-based treatment vs ibuprofen**												
4	randomised trials	serious[a]	very serious[b]	serious[c]	very serious[e]	none	202	212	-	MD **0.36 higher** (0.33 lower to 1.06 higher)	⊕◯◯◯ Very low[a,b,c,e]	CRITICAL
**pain intensity - plants-based treatment vs mefenamic acid**												
8	randomised trials	serious[a]	very serious[b]	serious[c]	very serious[e]	none	357	362	-	MD **0.06 lower** (0.4 lower to 0.29 higher)	⊕◯◯◯ Very low[a,b,c,e]	CRITICAL
**pain intensity - plants-based treatment vs placebo**												
13	randomised trials	serious[a]	very serious[b]	serious[c]	serious[d]	none	477	464	-	MD **1.83 lower** (2.32 lower to 1.34 lower)	⊕◯◯◯ Very low[a,b,c,d]	CRITICAL

CI: confidence interval; **MD:** mean difference

Explanations

a. The risk of bias of the studies is moderate.
b. The heterogeneity between the studies is very high.
c. The indirectness is due to the differences in the intervention treatments used across studies.
d. The sample size is small in each study, and the confidence interval is relatively narrow.
e. The sample size is small in each study, and the confidence interval is quite wide.
